# Viral Modulation of TLRs and Cytokines and the Related Immunotherapies for HPV-Associated Cancers

**DOI:** 10.1155/2018/2912671

**Published:** 2018-05-02

**Authors:** Marconi Rego Barros, Talita Helena Araújo de Oliveira, Cristiane Moutinho Lagos de Melo, Aldo Venuti, Antonio Carlos de Freitas

**Affiliations:** ^1^Laboratory of Molecular Studies and Experimental Therapy (LEMTE), Department of Genetics, Center of Biological Sciences, Federal University of Pernambuco, Av. Prof Moraes Rego, 1235 Cidade Universitária 50670-901 Recife, PE, Brazil; ^2^Laboratory of Immunological and Antitumor Analysis (LAIA), Department of Antibiotics, Center of Biological Sciences, Federal University of Pernambuco, Av. Prof Artur de Sá, s/n, Cidade Universitária 50740-525 Recife, PE, Brazil; ^3^Tumor Immunology and Immunotherapy Unit, Department of Research, Advanced Diagnostic and Technological Innovation, Regina Elena National Cancer Institute, Via Elio Chianesi 53, 00144 Roma, Italy; ^4^HPV-Unit, UOSD Tumor Immunology and Immunotherapy, Regina Elena National Cancer Institute, Via Elio Chianesi 53, 00144 Roma, Italy

## Abstract

The modulation of the host innate immune system is a well-established carcinogenesis feature of several tumors, including human papillomavirus- (HPV-) related cancers. This virus is able to interrupt the initial events of the immune response, including the expression of Toll-like receptors (TLRs), cytokines, and inflammation. Both TLRs and cytokines play a central role in HPV recognition, cell maturation and differentiation as well as immune signalling. Therefore, the imbalance of this sensitive control of the immune response is a key factor for developing immunotherapies, which strengthen the host immune system to accomplish an efficient defence against HPV and HPV-infected cells. Based on this, the review is aimed at exposing the HPV immune evasion mechanisms involving TLRs and cytokines and at discussing existing and potential immunotherapeutic TLR- and cytokine-related tools.

## 1. Introduction

**Figure 1:**
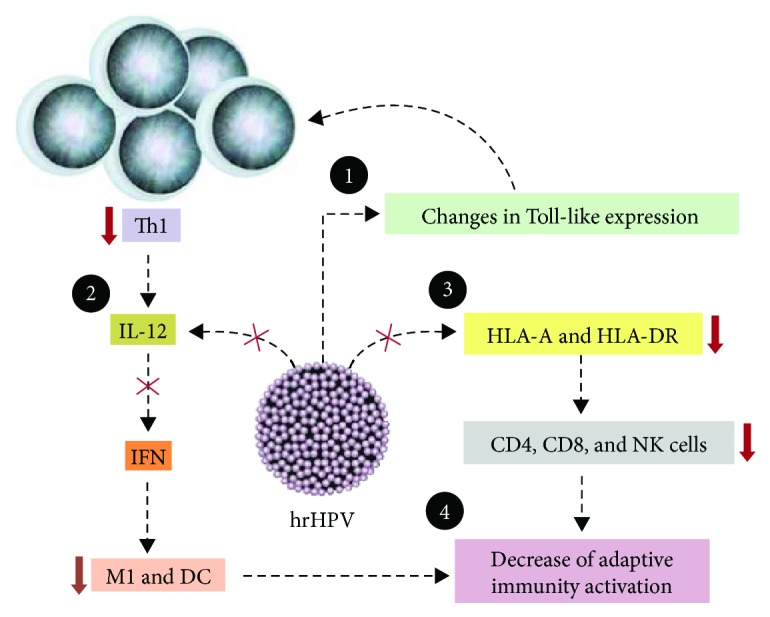
Th1 immune evasion induced by HPV in tumor microenvironment. HPV (1) interferes in TLR expression and in immune signalling pathways; (2) suppresses IL-12 expression, leading to a decreased production of IFNs (type I and IFN-*γ*) and blocking macrophages (M1 phenotype) and dendritic cell (DC) activity; (3) promotes downregulation of HLA expression and transportation to membrane surface, preventing antigen presentation, T cell activation, and NK and CTL cytotoxicity; and (4) finally, the downregulation of lymphocyte activity related to a decreased activity of APC cells (as M1 and DC) impairs adaptive immune activation [2].

The evasion of immune tumor surveillance is a well-established feature of cancer [1]. In HPV-related tumors, papillomavirus is responsible for this escape (Figure 1). This virus is able to abrogate the initial steps of the immune innate system, which embraces Toll-like receptor signalling as well as cytokine synthesis and secretion, thus, compromising the immune response against an invasive agent [2].

TLRs and cytokines play pivotal roles in the immune defence against HPV-infected and tumor cells. TLRs, for example, are responsible for recognizing the conserved pathogen-associated molecular patterns (PAMPs), promoting changes on host endogenous ligands and thus, initiating a protein cascade that follows in the expression of key molecules for the development of the immune response, which includes the synthesis and secretion of cytokines [3]. Cytokines, in turn, are important mediators of immune cell activities, such as cell recruitment, maturation, and signalling [4]. In addition, both molecules (TLRs and cytokines) control gene expression and are essential for creating a suitable tumor microenvironment, either for immune surveillance or for immune modulation. As a result, these molecules are involved in the pathogenesis of various diseases besides cancer, such as autoimmune, inflammatory, and infectious diseases [5].

Therefore, both TLRs and cytokines are crucial targets for immunotherapeutic studies that are aimed at preventing or treating cancer. In fact, they have already been used in pharmaceutical formulations for cancer therapies by taking advantage of the fact that currently there is no effective treatment for HPV-related cancer patients, especially for those who have unsuccessfully undergone radio- and chemotherapy treatments. Several major studies have reported the great potential value of immunotherapeutic approaches that modulated TLR and cytokine levels or synthesis [6, 7]. In accordance with these studies, this review highlights the immune mechanisms of TLRs and cytokines for infection resolution and viral immune evasion activities correlated with HPV-associated cancers. Furthermore, we will discuss the effectiveness of the immunotherapeutic approaches involving these targets.

## 2. Toll-Like Receptors

TLRs are specialized receptors which detect PAMPs and damage- or danger-associated molecular patterns (DAMPs). They are found in immune (e.g., APCs, natural killer) and nonimmune (e.g., stromal, epithelial, and cancer) cells on the plasma membrane (e.g., TLRs 1, 2, 4, 5, 6, and 10) and on the surface of organelles, such as endosomes, lysosomes, and endoplasmic reticulum (e.g., TLRs 3, 7, 8, and 9) [5].

The molecular structure of these receptors is comprised of two domains: the extracellular N-terminal and the intracellular C-terminal domains. The extracellular domain contains leucine-rich repeats responsible for recognition of PAMPs, depending on the TLR subtype; the C-terminal portion has a conserved region called Toll/IL-1 receptor (TIR) domain [5], which is responsible for transducing the signal for adapter molecules.

**Figure 2:**
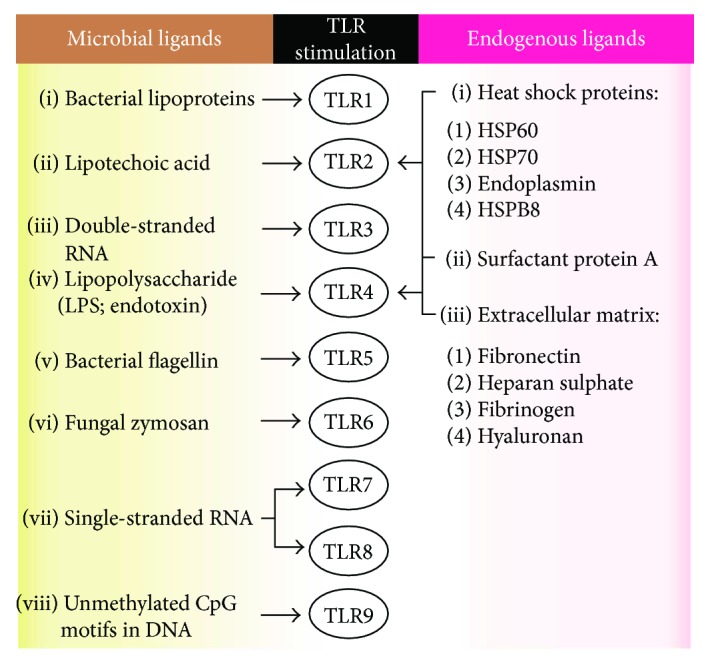
TLR activation. The scheme shows major microbial and endogenous ligands that activate TLR signals in immune cell surface. These signals are able to promote host protection against pathogen invasion and infection establishment [5].

Usually, TLRs are associated with the protection against pathogen invasion, carcinogenesis, and infection clearance, which are essential in inducing and linking innate and adaptive responses, such as the Th1 and the cytotoxic cell-mediated subtypes [3]. In addition, TLRs are able to recognize some host endogenous ligands (see Figure 2), representing an important role in tissue repair and homeostasis [9].

TLRs support the uptake, processing, and presentation of antigens by APCs, boost DC maturation, NK cell cytotoxicity, and targeted cell apoptosis as well as upregulate the expression of major histocompatibility complex (MHC), C-C chemokine receptor 7 (CCR7), interferons (e.g., IFN-I, IFN-*γ*), and inflammatory cytokines (e.g., IL-6, IL-12) [3]. Nevertheless, several TLRs were reported to be overexpressed in cancer. They were associated with malignant transformation by preventing the activation of immune responses or enhancing inflammation through the induction of the nuclear transcription factor kappa B (NF-*κ*B) pathway. Therefore, TLRs seem to have dual functions in the tumor microenvironment, to the extent that even cancer cells may express those molecules in order to alter immune response and sustain malignant progression [3, 10]. Indeed, recently, TLRs were showed to activate the nitric oxide signalling pathway, supporting cervical carcinogenesis [11].

The expression of both TLR4 and 9 receptors was reported to be altered during HPV infection. In cervical carcinogenesis studies, TLR4 and 9 levels were reported to be crucial for the initiation of the innate immune response due to the induction of cytokine synthesis and cytotoxicity on target cells. As a consequence, lower levels are generally associated with a poor prognosis and cancer progression [8, 12, 13]. However, these receptors have also been correlated with malignancy [3, 11, 14, 15]. TLR4 was found to be overexpressed in human cervical cancer line (HeLa) [11, 14] as well as in premalignant and malignant specimens [16]. It was also associated with the proliferation of cancer cells and immunosuppression, through the production of IL-6, TGF-*β*, and other immunomodulatory cytokines [3, 10]. TLR9 was also shown to be overexpressed in high-grade cervical lesions and/or cancer [3, 13, 15–17]. It is possible that the upregulation of these receptors in malignant lesions may be due to the following components: (i) compensation for TLR deficiency or by harnessing mechanisms of the host immune defence system against tumor cells, (ii) reflecting increased inflammation, which damages an effective immune response against the pathogen, (iii) the existence of polymorphisms and the measurement of different subtypes, which misleads data interpretation, (iv) the measurements at different intervals during cancer progression, (v) the differences in methods of TLR assessment in cell lines, or (vi) the activity of tumor cells that create a proper milieu for cancer development.

**Figure 3:**
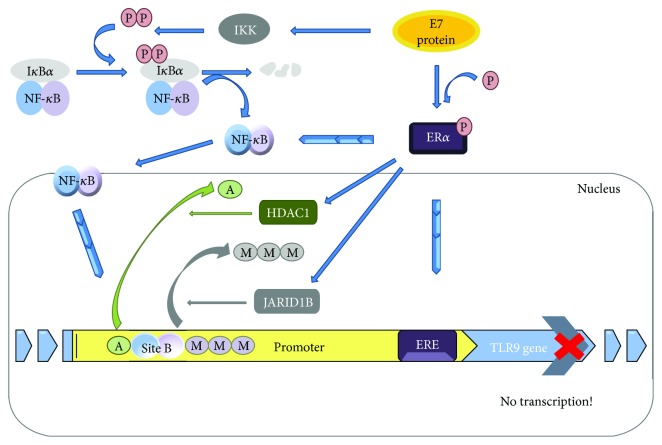
Inhibition of TLR9 expression by HPV16 E7 oncogene takes place via NF-*κ*B canonical pathway, when this oncoprotein recruits the inhibitory complex NF-*κ*B p50–p65 to a new *cis* element at the TLR9 promoter. This occurs with the additional binding of ER*α* (estrogen *α*) to another neighbour *cis* element, ERE (estrogen-responsive element), within that same promoter, and in the presence of HPV16 E7. ER*α* is also able to interact with the p65 subunit in the peri- or intranuclear region and contribute to transcription repression. Furthermore, it was also observed that there was a chromatin repressive complex composed by JARID1B demethylase and by HDAC1 deacetylase. These two catalytic units interact with ER*α* and negatively regulate TLR9 expression. The consequence of preventing TLR9 expression is the establishment of an immunosuppressive status with the inhibition of interferon and immune surveillance by cytokine responses [8]. NF-*κ*B blue circle corresponds to p50 subunit and the purple one to p65. The straight blue arrows indicate an activation process or progress to the next stage; the curved arrows indicate motion; and the progressive arrows indicate the movement of some molecules interacting with the target. IKK: inhibitor of kappa B kinase; P: phosphate group; M: methyl group; A: adenyl group; JARID1B: lysine-specific demethylase 5B; HDAC1: histone deacetylase 1; Site B: 9-10 base pair DNA sites where p50 and p65 subunits bind.

Modulation of TLR9 levels probably occurs due to HPV16 E7 oncoprotein activity on the TLR9 promoter by interference in the NF-*κ*B pathway. A chromatin repressive complex was also found on the TLR9 promoter, negatively regulating its transcription (Figure 3) [8]. A repression of the TLR9 expression disturbs the synthesis of IL-6, IL-8, and C-C chemokine ligand 20/macrophage inflammatory protein-3*α* (CCL20/MIP-3*α*) (which is an important chemokine for immune surveillance because of the recruitment of lymphocytes and Langerhans cells (LCs) to skin) [18]. The viral transcription and replication processes also undergo intervention, due to interferon deficiency caused by TLR9 repression [8]. Moreover, the altered levels of TLR9 can be caused by the devious expression of specific polymorphisms that change the effective receptor availability [19].

Other TLR expressions were also notably altered. In SCC (squamous cell carcinoma) specimens, it was demonstrated that the TLRs 3, 4, and 5 were significantly underexpressed while TLR1 was the only significantly overexpressed (TLRs 2, 7, 8, and 9 were not significant) when compared to the normal samples [13]. However, opposite results were also observed [3, 10, 11, 15, 16, 20]. No study has clearly demonstrated that altered TLR levels are a response of the host immune system against the infection or a consequence of virus activity supporting the infection. In addition, the knowledge of which cell is responsible for the alteration of TLR levels would be crucial to understand the TLRs' role in carcinogenesis; immune, stromal, and cancer cells have different functions in cancer development and thus, the shift of their TLR expression pattern could represent a marker for cancer progression or resolution [15].

Besides, another study found similar results and showed that the mRNA expression of TLR7 and 8 in cervical biopsies of cervical cancer patients was elevated [2]. In turn, infection regression (HPV16) was also associated with an increased expression of several TLRs (3, 7, 8, and 9), and their modulation could be used as a therapeutic approach [18].

Regarding other HPV-related cancers, TLRs were not as extensively studied like in cervical cancer. HPV is a key etiological factor of head and neck squamous cell carcinoma (HNSCC), in particular of oropharyngeal squamous cell carcinoma (OPSCC). HNSCC is commonly recognized as an immunosuppressive disease due to HPV activity. Consequently, an imbalanced cytokine profile, low amounts of CD3^+^ CD4^+^ and CD8^+^ T lymphocytes, high infiltration of M2 macrophages (TAM), Treg cells and Treg/CD8^+^ T cell ratio, impaired NK cell activity (higher expression levels of KIR genes), augmented expression of inhibitory receptors (e.g., CTLA-4, LAG-3, TIM-3, and PD-1), and decreased antigen presentation have been observed [21, 22].

Thereafter, the expression levels of several TLRs were reported and its dubious role was evident in some cases. It was found that TLR2 was upregulated in both vicinity immune cells and malignant keratinocytes in the microenvironment of oral squamous cell carcinoma (OSCC) compared to hyperplasia [23]. In another OSCC study, TLR3 was found to be upregulated in three head and neck cell lines (mRNA and protein), as well as when it induced apoptosis of the tumor cells, and *in vivo*, when it interrupted tumor growth. Also in this study, the mRNA expression of other TLRs was assessed observing elevated expressions of TLRs 1, 2, 4, and 6 while the TLRs 5, 7, 8, and 10 were found reduced [24]. In this type of carcinoma, however, TLR3 was found to have elevated levels and this event was attributed to an increase of tumor aggressiveness and invasion [25], a similar conclusion of another study which measured TLR3 in various HNSCC cell lines [26]. Both studies associated the triggering of the NF-*κ*B pathway with the increase in tumor aggressiveness. In the mentioned studies above, it was not verified whether HPV was present or not.

TLR4 and 9 were also assessed in OSCC, and these receptors were found to have elevated expression levels which were correlated with tumor development [10]. Whether the increased levels of these receptors were associated with carcinogenesis or a reaction of the host immune response against tumor cells is still not known. TLR4 was also correlated with tumor development and protection of cancer cells from host immune response in an HNSCC study [27]. Several other studies have also reported controversial results regarding OSCC and HNSCC [10, 28], though it has been observed that different cell lines were used several times for the same purpose, which could explain the opposite results in some cases.

TLR7 was found overexpressed in the nuclear membrane and nuclei of cancer cells (a novel localization discovered) having higher levels in HPV-positive specimens, unlike TLR9 that showed reduced expression levels in HPV-positive samples compared to HPV-negative. TLR7 also showed altered localization depending on the cancer cell status: it was found with elevated levels in the plasma membrane of cancer-free cells compared to cancer cells, where this receptor was observed to have higher levels in the nuclear membrane and nuclei. Therefore, it seems that TLR7 may play different roles depending on the cancer cell status. Furthermore, increased levels in TLR7 demonstrated to be statistically significant for the direct correlation with p16. Thus, the increased levels of TLR7 could be indirectly related to E7 expression since the elevated levels of p16 are caused by the deregulation of pRb pathway, initially caused by E7 oncogenic activity, which probably also altered TLR9 levels. The other TLRs did not show any significant differences between cancer and control groups [29].

Based on what has been discussed above, the modulation of TLR expression or activities has been considered in the treatment of cervical and head and neck carcinomas as adjuvants in various vaccine strategies. The goal is to increase the synthesis of cytokines (e.g., IFN, TNF-*α*) and chemokines, generally by activating NK and dendritic cells, so as to activate CTL cells for generation of effector responses [30]. For a more efficient outcome, TLR agonists can be used in combination with non-TLR agonists, as demonstrated in a C57BL/6 mice tumor model in which HPV16 E7 vaccine was coadministrated with monophosphoryl lipid A (MPL), a TLR4 agonist, and *α*-galactosylceramide [7]. Also, TLR activities can be blocked aiming to hamper inflammation by preventing TLR downstream activation signalling, such as the MyD88-NF-*κ*B pathway. Another interesting view to be highlighted is the importance of the modulation of TLR expression in stromal cells. It was found that these receptors were upregulated in these cells and may contribute to carcinogenesis [15].

Accordingly, several pharmacologic substances, which modify the activity of these receptors, have been tested/used first as adjuvants in vaccines for cervical cancer (e.g., Cervarix) and later in other HPV-related cancers. In the Cervarix vaccine for example, MPL is used to activate the innate response by interferon and proinflammatory cytokine synthesis. As a consequence, the adaptive response is also induced. Other examples are CpG (TLR9 agonist) alone or with rlipo (TLR2 agonist) [31], imiquimod and resiquimod (both TLR7 agonists), poly(I:C) (TLR3 agonist), and VTX-2337 (TLR8 agonist). Many studies have shown satisfactory results, especially with the simultaneous use of these agonists. Generally, an increased rate of tumor cell death upon TLR stimulation was observed [30]. Also, very high percentages of curing preexisting tumors in mice were reported [31–33].

In HPV-related cancers, the use of these adjuvants has also brought better outcomes. Imiquimod is able to induce Th1 responses by stimulating DC maturation and migration, Langerhans cell migration to draining lymph nodes, the inhibition of myeloid-derived suppressor cells, and the secretion of interleukins and cytokines [34]. In another study, imiquimod and poly(I:C) effects on cell death in *in vitro* and *in vivo* HNSCC models were evaluated. Both agonists were found to cause an increase in tumor cell death *in vitro* and *in vivo*. In addition, poly(I:C) induced higher levels of proinflammatory cytokine secretion (IFN-I, IL-6, and CXCL-10), MHC I expression of tumor cells, and monocyte activation *in vitro*, impairing tumor growth *in vitro* and *in vivo* even when TLR signalling was hampered on host cells [35]. Another study is currently recruiting participants to evaluate polyICLC (a modified version of poly(I:C)) combined with tremelimumab, a CTLA-4 antibody, and durvalumab, a PD-L1 antibody (clinicaltrials.gov identifier NCT02643303).

Another example is motolimod (VTX-2337), a TLR8 agonist which was tested for the treatment of HNSCC and other tumors. It was seen that this substance caused an increase in antitumor activities by inducing cytokine and chemokine synthesis as well as activating monocytes, NK, and dendritic cells, consequently boosting T cell activation [36, 37]. In a study using the TLR8 agonist with an anti-EGFR monoclonal antibody (cetuximab), an increase in NK cell-mediated cancer cell lysis and an enhancement of DC cross-priming of EGFR-specific CD8^+^ T cells were observed [38]. Other possibilities are currently being tested for VTX-2337, such as combining it with cisplatin or carboplatin/fluorouracil/cetuximab (NCT01836029) and with cetuximab or cetuximab and nivolumab, an anti-PD-1 antibody (NCT02124850). In regard to TLR4, OK-432 (picibanil, approved in Japan) was tested in association with DCs and chemotherapy, and the results still have not been reported (NCT01149902) [30].

**Figure 4:**
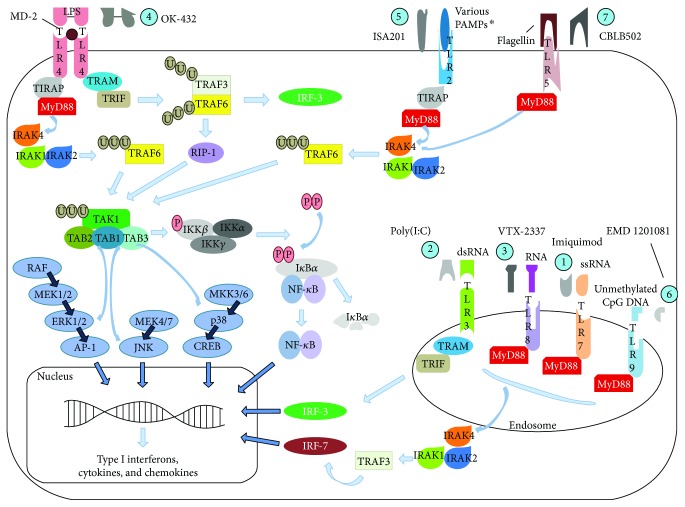
TLR signalling transducing pathways and the related immunotherapy approaches. Toll-like receptors are expressed in both immune and tumor cells, making difficult to decipher their function in cancer. Thus, their modulation in tumor microenvironment results from a complex balance of host and infected/tumor cell mechanisms which affects their expression and activation. They are activated by specific ligands (for each TLR) and synthetic substances as showed in the figure: (1) single-stranded RNA and imiquimod for TLR7, (2) double-stranded RNA and poly(I:C) for TLR3, (3) ssRNA and VTX-2337 for TLR8, (4) LPS, lipoteichoic acid, and Picibanil for TLR4, (5) ^∗^lipoproteins, peptidoglycans, lipoteichoic acids, zymosan, mannan, tGPI-mucin, and ISA201 for TLR2, (6) unmethylated dinucleotide cytosine-guanine and EMD 1201081 for TLR9, and (7) flagellin and CBLB502 for TLR5. Once activated, the signal transduction depends on adapter molecules such as MyD88, TIRAP, TRIF, and TRAM in order to activate the transcription of type I interferons and TLR-induced genes. All TLRs, except TLR3, require MyD88 for propagation of their signals. TLR4 requires myeloid differentiation factor-2 (MD-2) as a coactivator for its activation by LPS binding [3, 5]. TIRAP: TIR-domain-containing adaptor protein; MyD88: myeloid differentiation primary response protein 88; IRAK: IL-1R-associated kinase; TRAM: Toll receptor-associated molecule; TRIF: TIR-domain-containing adapter-inducing interferon-*β*; TRAF: TNF receptor-associated factor; RIP-1: receptor-interacting protein kinase-1; TAK: TNF receptor-associated factor; TAB: TGF-*β*-activated kinase I/MAP3K7-binding protein; IKK: inhibitor of kappa B kinase; I*κ*B*α*: nuclear factor of kappa B, alpha; IRF: interferon regulatory factor; U: ubiquitination; P: phosphorylation.

Other studies have also been or are currently being conducted with other TLRs: ISA201 (HESPECTA, a second generation vaccine based on ISA101), which uses a synthetic TLR2 agonist (Amplivant) with two HPV16 E6 peptides (NCT02821494) [38]; EMD 1201081 (IMO2055, TLR9 agonist) + cetuximab (NCT01040832), which did not demonstrate additional clinical efficacy than using cetuximab alone but it was well tolerated by patients [39]; EMD 1201081 + fluorouracil + cisplatin + cetuximab (NCT01360827); and entolimod (CBLB502), a TLR5 agonist which is being tested in combination with cisplatin and radiation (NCT01728480) due to the previously shown effects on radiation: it induced an increase in its therapeutic effect and a reduction of its toxicity *in vivo* when administered 1 h after exposure to radiation [40].  Table 1 summarizes the mentioned studies and is correlated with the Figure 4 which shows the therapeutic role and the activated signalling pathways of natural and synthetic TLR ligands.

## 3. Cytokines

Cytokines are the primordial mediators of the immune response, including antitumor activities. It has been reported that in cervical cancer as well as in HNSCC, immunostimulatory signals (Th1 cytokine profile) are hampered whereas proinflammatory and immunosuppressor ones (Th2 cytokine profile) are stimulated. Several studies have reported this shift of the cytokine pattern in preneoplastic and cancer specimens [41–43].

It is known that Th1 cytokines are potent activators of cellular-mediated immunity response that may precede HPV clearance, while Th2 cytokines impair the immune response, leading to an inefficient virus elimination and to chronic infection [43]. Furthermore, different hrHPV genotypes are associated with different cytokine profiles, so they interfere distinctly with the immune system, making disease progression different among the various hrHPV subtype infections [44]. Therefore, the modulation of cytokine expression is a key event for the induction of chronic infection and cancer development. It is known that the appropriate cytokine pattern defines the appropriate phenotype of immune cells, which whether or not results in the elimination of infected and (pre)cancerous cells.

Thus, cytokines were widely used in cancer immunotherapy, including cervical cancer and HNSCC. The main goal of their use was to induce a CTL response supporting the cancer cell apoptosis and tumor regression. They can be used in combination with several immunotherapy approaches such as DNA, DC-based and protein-based vaccines, TLR agonists, and monoclonal antibodies (e.g., cetuximab and the immune checkpoint inhibitors like tremelimumab and durvalumab) [6, 34]. In the next subheadings, the most important cytokines for the treatment of HPV-related cancers are discussed according to their roles in the immune response.

### 3.1. Immunostimulatory Cytokines

Among the immunostimulatory cytokines, IL-2, IL-12, TNF-*α*, and interferons are the most prominent in anti-infection and antitumor activity. IL-12 is secreted by activated DCs and macrophages and is the most effective and promising cytokine for cervical cancer treatment. Several antitumor activities in animal models have been observed [34], such as the increase in IFN-*γ* and TNF-*α* levels, maturation of APCs and the lysis of immature ones, and the activation of NK responses (caused by the upregulation of NK activation receptors and ligands). Consequently, IL-12 stimulates Th1 polarization and CTL (antigen-specific response) cytotoxicity and plays an important antiangiogenic role [45].

As a result, IL-12 has been suggested to be used in several HPV-related cancer treatment strategies for potentiation of antitumor activity, for instance, in viral gene therapy, coadministrated with other cytokines or in DNA vaccine preparations [34, 38, 45], such as the INO-3112 vaccine. This promising strategy, which combines the gene sequences of E6 + E7 antigens (VGX-3100) and the IL-12 (INO-9012), has been tested for treatment of both cervical invasive (NCT02172911) and head and neck (NCT02163057) HPV-related cancers in clinical phase I/II trials with good results about safety and CD8^+^ T cell immunogenicity [46].

Another ongoing study evaluated the immunotherapeutic effects of the coadministration of the recombinant IL-12 with cetuximab (NCT01468896) in patients with relapse, metastatic, or inoperable HNSCC. In the previous study regarding this combined treatment, an improvement in the lysis of tumor cell by NK cells was observed. In another approach using IL-12, a higher lymphocyte infiltrate and an improved overall survival rate were observed when IL-12 was coadministrated with IL-1*β*, IFN-*γ*, and TNF-*α* [47].

TNF-*α* is another key cytokine which creates an antitumoral milieu for virus elimination. This molecule supports the activation of macrophages, dendritic and NK cells and recruits them to the tumor site by inducing keratinocytes to release MIP-3*α* [18] and CCL2/MCP-1 (C-C motif chemokine ligand 2/monocyte chemoattractant protein-1), which is reduced in high-grade cervical lesions and in E6/E7-expressing cells [48]. Moreover, it inhibits HPV E6 and E7 oncogene transcription and proliferation in HPV-transformed keratinocytes *in vitro* [49] and causes apoptosis of cervical cancer cell lineage [50]. This cytokine is associated with lesion regression; its reduced level in serum and its presence in cervical cancer are correlated with tumor growth [51, 52]. Increased levels of TNF-*α* were also associated with CIN2/3 lesions, but also with an exacerbated inflammatory response [18]. A precise level of inflammatory response, which includes the precise secretion level of several cytokines including TNF-*α*, is the threshold between the occurrence and regression of cellular transformation. A sufficient level of this response at the infection onset is a mark of a valid immune response, but when the inflammatory response becomes excessive and persistent, it favours an appropriate milieu for infection development.

Along with TNF-*α*, interferon belongs to the group of crucial cytokines which creates an antiviral state, activating cell-mediated immunity which is potentiated in the presence of TNF-*α* (see Table 2 for IFN activities). Interferon is classified in type I (IFN-*α* and -*β*), type II (IFN-*γ*), and type III (IFN-*λ*) [62]. Similar to TNF-*α*, the amount of interferon arises mainly from NK cells [63], but T cells also synthesize these cytokines, both cytokines are upregulated in therapeutic approaches which cause infection regression [34].

With regard to this, it was suggested that the upregulation of IFN would be a good hallmark for HPV16 infection clearance [64]. This event is generally associated with the expansion of NK cell cytotoxicity [65] and the expression of IL-12 and TNF-*α* in infiltrated proinflammatory lymphocytes [57]. The increased levels of IFN restore immune function [55] and induce CD4^+^ and CD8^+^ responses, which led to a complete regression of the disease in a half of the patients who had undergone treatment. Several reports have shown the inhibition of type I IFN expression or their transduction pathways by HPV16 and 18 oncoproteins (E6 and E7). In this way, key genes involved in the immune surveillance and cytotoxic response are blocked [2, 56].

Diminished IFN-*γ* synthesis has also been associated with persistent infection and the level of malignancy of cervical lesions [66] as well as to the development of HPV-related cancer. In HNSCC, for example, the reduced levels of this cytokine are able to modify STAT1/STAT3 balance that blocks antigen presentation and DC maturation [47].

IFN-*γ* became a marker of the Th1-type antigen-specific cellular immune responses, involved in tumor resolution [67] and HPV clearance [18]. This cytokine induces: (i) synthesis of antiangiogenic factors (e.g., IP-10); (ii) cell cytotoxicity by boosting the overexpression of adhesion molecules; (iii) antigen presentation and synthesis of IL-12 by activation of DC; and (iv) more sensitivity to granule activity and death signalling [68]. In addition, type III IFN (IFN-*λ*) has also been demonstrated to play an important role in immune response against HPV infection. Alteration of this interferon was reported in hrHPV cell infection status, and it was suggested that it may support immune surveillance against HPV infection and cervical carcinogenesis. IFN-*λ* shows similar activities of type I IFNs but only specific cells respond to this type of IFNs like epithelial cells [62]. For all these activities and owing to the oncoprotein interference with these molecules, interferons are a class of cytokines with a great potential value when used in the development of more efficient approaches for HPV-related cancer therapy.

Another cytokine demonstrating to have strong antitumor activity is IL-2, which is produced primarily by CD4^+^ cells after antigen priming. It plays several important roles, including the activation and maturation of DC, stimulation of NK cell cytotoxicity, the expansion of CD8^+^ and CD4^+^ cells, and the polarization for the Th1 cell profile [69]. Several studies have attributed the reduced levels of this cytokine to the lesion progression or cancer in HPV infection [21, 42, 70, 71]. Its reduced levels were suggested to be a viral evasion mechanism [71], which is frequently associated with increased levels of IL-10, TGF-*β*, and Treg cells [43]. Due to its strong immunoprotective activity, IL-2 has been used in several immunotherapeutic approaches, like in association with TG4001 HPV vaccine, which is constituted of a recombinant Ankara vaccinia virus expressing HPV16 E6 and E7 [6]. The disadvantages of IL-2 administration are systemic toxicity and the induction of Treg cell proliferation [69]. For example, in HNSCC, the administration of IL-2 and IFN-*α*2a demonstrated high toxicity [72].

### 3.2. Th17 Cells and Proinflammatory Cytokines

The inflammation process is an important feature of any cancer [1]. In regard to HPV-associated cancer, a deregulated proinflammatory network is induced by HPV inducing a favourable milieu for tumor development.

Th17 cell (CD4^+^, IL-17^+^), a T cell phenotype involved in the inflammatory response, was reported to be linked to the development of cervical cancer as well as others [73]. It has been shown that the percentage of this cell phenotype, as well as Th17/Treg ratio, was higher in peripheral blood samples of patients with premalignant and cervical cancer lesions when compared to the normal cytology group [74], a similar outcome previously observed [75]. In other studies, elevated levels of Th17 cells in CIN and cancer patients [73, 76] and in high-grade cervical lesions [77] were found, when compared to healthy controls. A higher statistical prevalence of this cell type was reported not only in serum but also in the cervical tissue of cancer patients. Likewise, a statistical significance of Th17 prevalence among patients with advanced (higher prevalence) and early stages in malignant processes was observed [78] and thus, its level has been deemed a good independent prognostic factor in cervical cancer [79].

In HNSCC, elevated levels of Th17 cells were found along with Treg [80] in serum and in tumor milieu, concluding its negative impact on the immune response against HPV, especially due to the induction of an exaggerated inflammatory response through IL-17 secretion [81]. The increased levels of Th17 or IL-17 were also found in patients with hypopharyngeal carcinoma, as well as other cancers such as colon, gastric and lung, suggesting a connection with cancinogenesis and malignant progression. Conversely, premalignant oral lesion treatments with TGF-*β* type I receptor inhibitor and IL-23 showed maintenance of the Th17 phenotype instead of changing to Treg cells. This resulted in the production of stimulatory and inflammatory molecules and slowed the progression of premalignant lesions to oral cancer [82]. The last research outcome demonstrated that the effect of Th17 is still unclear, mainly in other types of HPV-related cancers [79].

IL-1 (both IL-1*α* and -*β*) is the other interleukin present in higher levels in cervical cancer. Secretion of IL-1 is promoted by keratinocyte damage, the major IL-1-producing cells [83]. This interleukin is also secreted by TAM, another important cell in harmful proinflammatory response which induces metastasis, tumor growth, angiogenesis, and Treg differentiation [21]. IL-1 expression is modulated by NK-*κ*B and vice versa, and the same occurs with TNF-*α*, that participates in the IL-1 synthesis pathway [83].

In particular, IL-1*β* exhibits an essential role in inflammation-associated carcinogenesis and supports tumor growth and metastasis. This interleukin promotes the secretion of a great range of cytokines, chemokines, growth factors, and various metastatic mediators, such as TGF-*β*, VEGF, metalloproteinases, and endothelial adhesion molecules [84]. In cancer studies, IL-1*β* is associated with a poor prognosis [84], and in cervical cancer research, it supports tumor progression and carcinogenesis [85]. IL-1*β* was also overexpressed in several types of tumors like breast, colon, oesophageal, lung, and oral cancer. A high throughput bioinformatics analysis plus *in vitro* and *in vivo* observation demonstrated that IL-1*β* is one of the key genes involved in HNSCC formation. This interleukin is closely related to the malignant transformation of oral cells, protumorigenic microenvironment generation that leads to oral carcinogenesis, and cell growth of the same type of cells [86]. Due to its crucial role in inflammation and carcinogenesis, this interleukin has been considered useful in therapeutic strategies [84] as well as IL-1*α* which also plays an important role in carcinogenesis [87]. Interestingly, the expression of IL-1*α* has been associated with higher risk of distant metastasis in HNSCC, the major cause of death in this type of cancer. In this scenario, the evaluation of IL-1*α* and clinical information may predict patients with high risk of HNSCC metastasis, thus leading to new treatment strategies [88].

Other examples of proinflammatory cytokines which support inflammation-associated cervical carcinogenesis are listed in Table 3.

### 3.3. Immunosuppressive Cytokines

There are several cytokines which are directly involved in downregulation of inflammatory status, promoting infection progress and cancer development. These include TGF-*β*, IL-4, IL-6, and IL-10 which are the main Th2 cytokines related to this anti-inflammatory profile and are discussed in this topic.

The expression of these cytokines is modulated by HPV oncoproteins o create a Th2 microenvironment. They have been reported to be upregulated in premalignant and cancer lesions [41, 52], and were suggested as biomarkers for HPV-related cancer [96]. Other Th2 interleukins are also encountered at high levels in cervical cancer patients, such as IL-9 [97] and IL-15 [44]. The latter and TGF-*β*, for example, were reported to induce the expression of CD94/NKG2A, preventing NK cell activity and CTL cytotoxicity [98]. An ongoing study evaluated a recombinant human IL-15 in advanced HNSCC patients in order to measure NK cell count, activity, and other immune response parameters (NCT01727076).

TGF-*β* plays a crucial role in the repression of immune responses against HPV and is upregulated in cervical carcinogenesis by viral oncoprotein activities. It blocks effector functions by suppressing antigen presentation, NK cytotoxicity, B cell and CTL proliferation, and cytokine synthesis of the Th1 profile. It downregulates IL-2 receptor signalling in T cells and IL-12 expression by APCs. Moreover, this cytokine is able to promote IL-10 expression by macrophages, induces proteolytic activity, which causes angiogenesis and metastasis [43], induces CD94/NKG2A expression on T cells [98], and stimulates the differentiation of T cells to Treg and Th17 phenotypes [74]. Moreover, TGF-*β* upregulation has been associated with favouring tumor development, CIN III specimens, cervical cancer, and cancer invasiveness [43].

IL-4 is another important cytokine frequently mentioned in cervical cancer studies. It is able to inhibit cytotoxic activity and IFN-*γ* expression, even in the presence of PMA (phorbol myristate acetate) and ionomycin—substances used in carcinogenesis models that stimulate immune responses [99]. This interleukin induces a switch to a Th2 responsiveness profile along with IL-2 [100] and is associated with viral persistence, disease severity, and progression of precancerous lesions [43, 100].

Like IL-4, IL-6 is upregulated during cervical carcinogenesis progression [90], playing a role in HPV-immortalized and carcinoma-derived cervical cell line proliferation [101]. It has been stated to induce the phosphorylation of STAT3 (the activated condition) in HNSCC, causing immunosuppression by inhibiting maturation of DC and activation of neutrophil, macrophage, NK and T cells. STAT3 is a key transcription factor which is involved in several other immunosuppressive activities such as IL-10 signalling, downregulation of IL-12, impairment of DC, and production of Treg cells [102]. IL-6 also contributes to the proliferation and inhibition of apoptosis of cancer cells, and thus, supports chronic inflammation and cancer development [51]. This interleukin affects DC migration, induces angiogenesis [103], MMP9 synthesis, and TAM differentiation [104]. However, IL-6 has also been reported to play anti-infection and antitumor functions [105]. Its expression is associated with a poor clinical prognostic factor [73] and its transcription repression can be used in immunotherapy approaches in cervical [106] and other HPV-related cancers [47].

IL-10 is the most studied Th2 cytokine with immunosuppressive activity in HPV-related cancer. It is synthesized by various cells, including Treg, Th2, M2 macrophages, APCs, and NK cells. This interleukin supports the creation of a microenvironment favourable to tumor development and it is the main cytokine having a Th2 role along with TGF-*β*. It hampers immune surveillance by blocking (i) antigen presentation by DC through the reduction of MHC II, adhesion, and costimulatory molecules, (ii) the synthesis of cytokines of the Th1 profile and (iii) the activities of monocytes and NK cells. IL-10 also supports immunomodulation by inducing the differentiation of T cells and macrophages to the Treg [43] and M2 profiles, respectively [107].

Furthermore, it has been demonstrated that IL-10 prejudices antiviral immune responses, since it impairs Th1 profile differentiation, CD8^+^ cytotoxic response, and CD3^+^ expression, which is essential in activating T cells. This interleukin also causes the downregulation of MHC I and II on the surface of monocytes. Moreover, this molecule is able to upregulate HPV16 E7 in cervical carcinoma cells *in vitro*, inducing tumor proliferation [43].

Despite the immunosuppressive activities cited above, there were disagreements over what high levels of IL-10 were related to, since it has been also associated with low grade lesions [66]. However, its immune regulatory activities have been well established. Elevated levels of IL-10 are commonly correlated with high-grade lesions and cancer condition, and its immunosuppressive roles have been reported countless times [42, 43, 52, 91], what are probably induced by HPV E2 protein activity [43].

**Figure 5:**
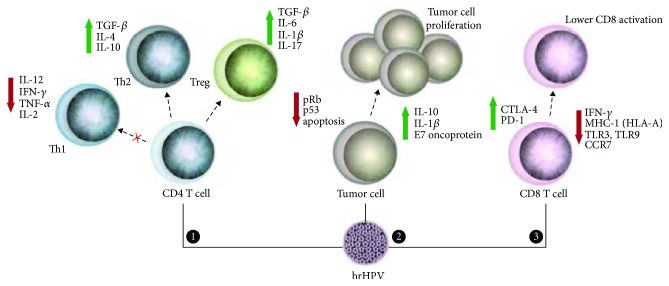
Interferences of HPV on CD4^+^/CD8^+^ lymphocyte responses, tumor proliferation, and apoptosis. (1) HPV is able to downregulate the Th1 response by decreasing proinflammatory cytokines. Moreover, Th2 and Treg response are stimulated by the virus because anti-inflammatory cytokines are expressed in higher amounts in tumor microenvironment. (2) Tumor cells show different mechanisms for their activation and survival. HPV (especially E6 and E7 oncogenes) promotes changes in cell cycle regulatory genes (e.g., pRb and p53), leading to tumor cell proliferation. In addition, Th2 cytokines produced by APC and T CD4^+^ cells generate a chemical microenvironment that favours tumor establishment [2]. (3) T CD8^+^ lymphocyte activities are affected by HPV infection that induces a decreased antigen presentation (MHC-I/HLA-A) and expression of TLRs and CCR7 on membrane surface of infected cells. Moreover, these cells also show upregulation of CTLA-4 and PD-1 inhibitor molecules [108].

Hence, IL-10, along with CD8^+^ T cells and Treg cell ratio, has been considered an independent factor of poor prognosis. Treg cell is associated with tumor growth, tumorigenesis, and lymph node metastasis, constituting a poor prognosis for patients with cervical and other cancers as well [55]. These cells are rich in high-grade carcinoma samples, suppress CTL and NK cell activities, and support cancer progression through cytokine synthesis, such as IL-10 itself and TGF-*β* [43, 80]. Therefore, Treg cells, IL-10, and TGF-*β* (and other immune activities interfered by HPV are summarized in Figure 5) are considered targets for therapeutic interventions.

Another cytotoxic cell has also been encountered at high numbers in cervical cancer samples [109]. The expansion of this novel T cell subtype might affect lesion fate [110]. It is positive for CD4 and NKG2D markers and is subdivided in CD28^+/−^, showing a statistically significant association with underexpression of several proinflammatory cytokines, such as IL1-*β*, IL-2, and TNF-*α* [109]. The role it plays and whether it is related to tumor growth or tumor suppression are still not known.

Novel cytokines in cervical cancer study have been gaining more and more attention due to their therapeutic potential. One of them, IL-37, has shown promising results because of its anticancer activity. For the first time, its anticancer role in an *in vitro* cervical cancer model has been demonstrated. This interleukin suppressed proliferation and invasion of HeLa and C33A cell lines, with higher inhibition rates in HeLa cell line, showing that anticancer activities were related to the HPV. This occurred through the inhibition of mRNA and protein expression of STAT3. Moreover, STAT3 phosphorylation was also blocked. This protein is a key transducer signalling molecule for developing an immune response in a tumor setting and could be an antagonist of IL-6 activity since STAT3 is central to the IL-6 signalling pathway [111].

In summary, cytokines are key molecules which modulate the pathology milieu. Thus, the regulation of the transcription, the synthesis, and the secretion of these signalling molecules in immunotherapies are necessary events to achieve satisfactory results in therapy. Cytokines are involved in several mechanisms of the immune system, such as immune cell maturation/differentiation, maintaining activation and regulating immune cell activities, which contribute to an immunoprotective background. Consequently, these molecules are important for therapy, i.e., by supporting the establishment of a proimmune milieu for infection clearance or by preventing the immunosuppressive role of immune cells. Many therapeutic interventions which take advantage of this have been developed and are currently in progress in therapeutic practices with very promising results, such as the active cytokine components of IRX-2, which caused an increase in antigen presentation, in NK cell activity, in the synthesis of costimulatory molecules and in CD8^+^ T cell responses [112]. The mixture of cytokines is safe, generally well tolerated, and has currently been tested in a phase II trial for HNSCC [38].

## 4. Future Prospects

The immune response is vital in HPV-related cancer disease progression and resolution. In this set of collected data, the host immune response was observed to be used to benefit patient's health through various ways, as these attributes occur in the natural resolution of infection. These new immunological approaches open novel horizons in diagnosis and especially in cancer therapy. TLRs and cytokines have been used to create an ideal tumor milieu for preventing tumor development or favouring transformed cell destruction. Their utilization appears to be a very promising immunotherapeutic strategy nowadays. The activation of DC and NK cells by means of administration of appropriate TLR and cytokines is essential to ensure the T-helper and cytotoxic responses.

Another possibility of using TLRs and cytokines in immunotherapy is their use in combination with monoclonal antibodies that prevent the activity of the immune checkpoint molecules, such as CTLA-4, PD-1, LAG-3, and TIM-3. These receptors are found in T cells and interact to ligands located at the cell membrane of tumor and antigen-presenting cells. In tumor pathogenesis, the activation of these molecules triggers signalling pathways which primarily prevent the function of CD8^+^ and CD4^+^ cells. In addition, the induction of other immune evasion activities offers a suitable environment for infection persistence and tumor development. Thus, inhibition of these activities may be considered a good therapeutic target; CTLA-4, for example, is found to be highly expressed in HPV-related cancer when compared to HPV-negative samples [22]. It competes with CD28 for interactions with the CD80 and CD86 ligands in DC surface, which prevented T cell priming by these cells. PD-1/PD-L1 is also greatly related to immune escape in HPV-related cancers, being correlated with a reduced disease survival rate due to CTL activity attenuation [102, 113]. Similarly, LAG-3 and TIM-3 have been recently considered in immunotherapy approaches. The first molecule enhances Treg cell activity [102]; and the second one induces T cell exhaustion and suppression of the innate response, associated with poor prognosis and tumor progression [114].

Checkpoint inhibitor monoclonal antibodies combined with other immune approaches have already been evaluated for cervical and HNSCC treatment: anti-PD-1 such as pembrolizumab (which is in ongoing studies for HNSCC treatment: NCT02255097 and NCT02252042) and nivolumab (NCT02105636 and NCT02488759); anti-PD-L1 such as durvalumab (NCT02207530) [46]; anti-CTLA4 such as ipilimumab and tremelimumab; anti-LAG-3 such as BMS-986016 (NCT01968109); and anti-TIM-3 (NCT02817633). The cited studies including LAG-3 and TIM-3 were for treatment of solid tumors. On the other hand, the combination with TLR agonists is new for HPV-related cancers; there are only two studies, NCT02643303 and NCT02124850, which are currently recruiting patients for testing the poly(I:C) with tremelimumab and durvalumab and the combination of VTX-2337 plus cetuximab or VTX-2337 plus cetuximab plus nivolumab, respectively. Regarding the evaluation of the combination of checkpoint inhibitors with cytokines in HPV-related cancers, no current study is reported at least to the best of our knowledge; and only three studies were reported for other HPV-unrelated solid tumors (NCT02614456, NCT02174172, and NCT02947165).

Therefore, the combination of different immunotherapeutic methods has shown increased beneficial effects and seems to be crucial in achieving better outcomes as observed in preclinical and clinical trials. As discussed here, HPV-related tumors require a great immune suppressor status for cancer development with increased activities of Treg, CTLA-4, and PD-1 and the suppression of APC and NK cells. Thus, studies on such evasion mechanisms are needed and offer new therapeutic perspectives.

## Figures and Tables

**Table 1:** TLR-associated immunotherapy approaches for HNSCC treatment.

Number in Figure 4	Therapy	Therapy approach	Stage	Clinical trial identifier	Reference
1, 2	Imiquimod + poly(I:C)	TLR7 agonist + TLR3 agonist	*In vitro*/*in vivo*	—	Klein et al. [35]

2	Poly(I:C)	TLR3 agonist + tremelimumab + durvalumab	Phase I/II	NCT02643303	No study was reported yet.

3	VTX-2337	TLR8 agonist	Phase I	NCT00688415	Dietsch et al. [37]
		TLR8 agonist + cetuximab	Phase I/II	NCT01334177	Stephenson et al. (2013) and Chow et al. [36]
		TLR8 agonist + cisplatin or +carboplatin/fluorouracil/cetuximab	Phase II	NCT01836029	No study was reported yet.

4	OK-432 (Picibanil)	TLR4 agonist	Phase I	NCT01149902	Galluzzi et al. [30]

5	ISA201	TLR2 agonist + 2 HPV16 E6 peptides	Phase I	NCT02821494	Bann et al. [38]

6	EMD 1201081	TLR9 agonist which was tested with cetuximab	Phase II	NCT01040832	Ruzsa et al. [39]
		TLR9 agonist + fluorouracil + cisplatin + cetuximab	Phase I	NCT01360827	No study was reported yet.

7	CBLB502 (entolimod)	TLR5 agonist	Phase I	NCT01728480	Toshkov et al. [40]

**Table 2:** IFN immune activities and HPV interferences.

IFN-I (IFN-*α*/*β*)	*Activities*
	IFN-*α* inhibits keratinocyte immortalization induced by HPV16 [53]
	Boosts IFN-*γ* secretion [54] and Th1 response [55]
	Induces antibody production [54]
	Induces resistance to viral replication [56]
	Induces MHC class I and II expression [54]
	Induces the activation of NK cells [56]
	Plays antiangiogenic and antiproliferative activities [57]
	Induces DC maturation and T cell proliferation and priming [54]
	Turns virus-infected cells more susceptible for CTL killing [56]
	Alters the B cell isotype and differentiation into plasma cell [54]
	Prevents T cell apoptosis [54]
	Promotes the proliferation of memory T cells [54]
	*HPV interferences*
	Its signal transduction pathways are prevented by E6 and E7 activities [18]
	IFN-*α* signalling is inhibited by (i) HPV18 E6 interaction with Tyk2 (tyrosine kinase 2), (ii) E6 binding to IRF-3 which prevents IFN-*α* transcription, and (iii) HPV16 E7 prevention of the displacement of p48 (subunit of interferon-stimulated gene factor 3 (ISGF3)) to the nucleus
	HPV16 E7 also inhibits IRF-1-mediated IFN-*β* transcription by physically interacting with IRF-1
	IFN-*α* and IFN-*β* are also downregulated by E6-mediated inhibition of STAT1 binding to ISRE and prevention STAT1 expression [57]

IFN-II (IFN-*γ*)	*Activities*
	IFN-*γ* as well as IFN-I inhibit transcription of E6/E7 genes in immortalized keratinocytes and malignant cells [48]
	Upregulates MHC class I in immune and tumor cells [58] as well as MHC I/II in epithelial cells [18]
	Promotes differentiation to the Th1 profile and induces these cells to produce IFN-*γ* [59]
	Plays antiviral, antiproliferative [58], and tumor cell killing activities, being associated with lesion regression [60]
	Boosts the synthesis of inducible nitric oxide synthase, IP-10 (protein 10 inducible by IFN-*γ*) and Mig (monocin inducible by IFN-*γ*) chemokines by macrophages, which augment cytotoxic response [58]
	Induces NK cell infiltration and activation [58]
	Induces TAP-1 (transporter antigen processing-1) and MCP-1 expression, which are important for T cell antigen recognition and chemoattraction, respectively [58]
	*HPV interferences*
	The expression of TAP-1 and MCP-1 is prevented by E7 and E6/E7, respectively [48]

IFN-III (IFN-*λ*)	*Activities*
	Prevents several tumor cell lines growth [61]
	Promotes antiviral and antitumor responses [61]
	In the treatment of viral and neoplastic diseases it has been tested with type I IFNs showing synergic effects and reduced side effects [61]
	*HPV interferences*
	None reported

**Table 3:** Some proinflammatory cytokines in HPV-related carcinogenesis.

Cytokine	Action mechanism
IL-8	(i) It induces neutrophil chemoattraction and cell survival [18] (ii) It stimulates cell growth and cancer metastasis [89] (iii) Prognosis of patients with high levels of IL-8 is extremely poor [48] and its expression was associated with lesion severity [90]

IL-17	(i) It is associated with lymphatic metastasis [91] (ii) It is found in high levels in patients with cervical cancer [91] (iii) It is also linked to the antitumor response [92], when it supports the recruitment and activation of neutrophils, the maturation of DC/priming of T cells, and the synthesis of TNF-*α*, IL-1*β*, and IL-6 [74, 75, 93]

IL-23	(i) It is synthesized by activated APCs [94] (ii) It induces macrophage secretion of TNF-*α*, DC production of IL-12, and the synthesis of IL-17 [95] (iii) It induces upregulation of MMP9 (matrix metalloproteinase 9), tumor angiogenesis, and TAM activity and prevents T CD8^+^ cell infiltration [95] (iv) As well as IL-17, shows antitumor activities through its immune surveillance properties, such as the promotion of CTL and NK cell, the IFN-*γ* secretion, and the stimulation of the IL-12-induced Th1 response [95]

## Abbreviations

APC: Antigen-presenting cell
CCL2/MCP-1: C-C motif chemokine ligand 2/monocyte chemoattractant protein-1
CCL20/MIP-3*α*: C-C motif chemokine ligand 20/macrophage inflammatory protein-3*α*
CCR7: C-C motif chemokine receptor 7
CTL: cytotoxic T lymphocyte (CD8 T lymphocyte)
CTLA-4: Cytotoxic T lymphocyte-associated antigen 4
DAMP: Damage- or danger-associated molecular patterns
DC: Dendritic cell
EGFR: Epidermal growth factor receptor
HDAC1: Histone deacetylase 1
HeLa: Human cervical cancer cell line
HNSCC: Head and neck squamous cell carcinoma
HPV: human papillomavirus
I*κ*B*α*: Nuclear factor of kappa light polypeptide gene enhancer in B-cell inhibitor, alpha
IRAK: IL-1R-associated kinase
IRF: Interferon regulatory factor
ISGF3: Interferon-stimulated gene factor 3
ISRE: Interferon response element
IKK: Inhibitor of kappa B kinase
JARID1B: Lysine-specific demethylase 5B
LC: Langerhans cell
LPS: Lipopolysaccharides
MHC: Major histocompatibility complex
MMP9: Matrix metalloproteinase 9
MPL: monophosphoryl lipid A
MyD88: Myeloid differentiation primary response protein 88
NF-*κ*B: Nuclear transcription factor kappa B
NK: Natural killer
OPSCC: Oropharyngeal squamous cell carcinoma
OSCC: oral squamous cell carcinoma
PAMPs: Pathogen-associated molecular patterns
PD-1: Programmed death-1 receptor
PD-L1: Programmed death ligand-1
PMA: phorbol myristate acetate
Poly(I:C): Polyriboinosinic-polyribocytidylic acid
PolyICLC: Modified version of poly(I:C) stabilized by lysine and carboxymethylcellulose
RIP-1: Receptor interacting protein kinase-1
SCC: squamous cell carcinoma
TAB: TGF-*β*-activated kinase I/MAP3K7-binding protein
TAK: TNF receptor-associated factor
TAM: Tumor-associated macrophage (M2 macrophage phenotype)
TAP-1: Transporter antigen processing-1
TIRAP: TIR-associated protein
TNF-*α*: Tumor necrosis factor *α*
TLR: Toll-like receptor
TRAM: Toll receptor-associated molecule
TRAF: TNF receptor-associated factor
TRIF: TIR-domain-containing adapter-inducing interferon-*β*.
